# “Slight chemical damage due to drinking modest amount of sake, might induce beneficial effects” as a form of hormesis: an interview with Professor Sataro Goto

**DOI:** 10.1007/s10522-023-10069-4

**Published:** 2023-10-26

**Authors:** Zsolt Radak

**Affiliations:** Hungarian University of Sport Science, Alkotas ut 42-46, Budapest, 1023 Hungary

**Keywords:** Toho University, Error theory, Protein modifications, Calorie restriction, Hormesis

## Abstract

Professor Sataro Goto is one of the pioneers of biological aging research in Japan. He is renowned for his work on the role of protein errors and modifications, the accumulation of abnormal proteins due to reduced protein turnover, and the modulation of aging and lifespan by adult-onset dietary restriction and regular exercise. Professor Goto is a remarkably intelligent, visionary, empathetic, humble, and wise man, who kindly agreed to this interview that I (Zsolt Radak) made with him during one of my frequent visits to his labs, in February 2023.

## Introduction

Professor Sataro Goto has been one of the pioneers of biological aging research in Japan. He has more than four decades of active and successful involvement in research, mentoring and promotion of aging research, especially during his tenure at the Toho University, Funabashi, working for many years as the head of the Department of Biochemistry and the Dean of the Faculty of Pharmaceutical Sciences. He is internationally renowned for his work on the role of protein errors and modifications, the accumulation of abnormal proteins due to reduced protein turnover, and the modulation of aging and lifespan by adult-onset dietary restriction and regular exercise. I had the great privilege of working with him first as a postdoctoral fellow (1998–2020), and, since then, continuing to associate and collaborate with him. Goto-san is a remarkably intelligent, visionary, empathetic, humble, and wise man, who kindly agreed to this interview that I made with him during one of my frequent visits to his labs in February 2023.
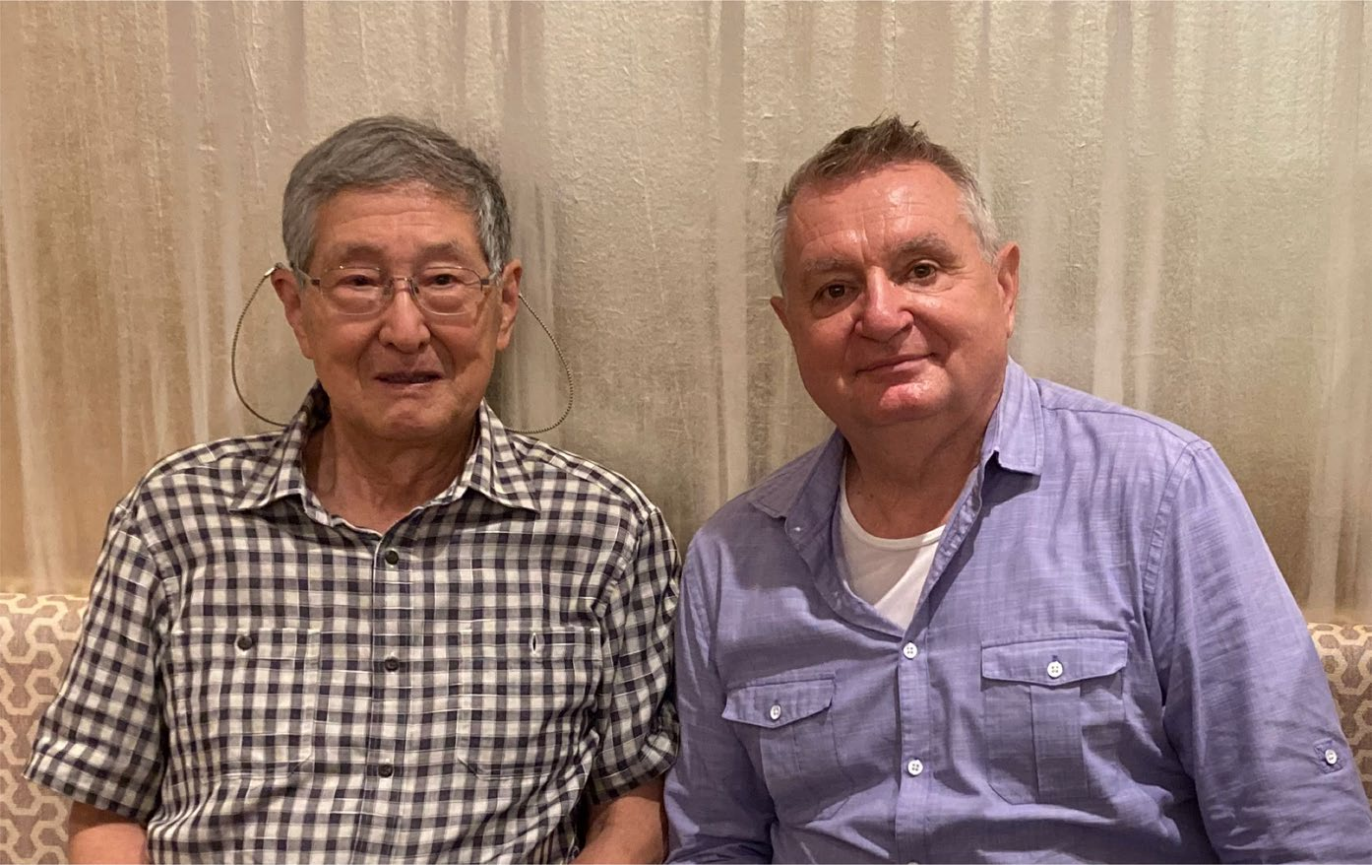

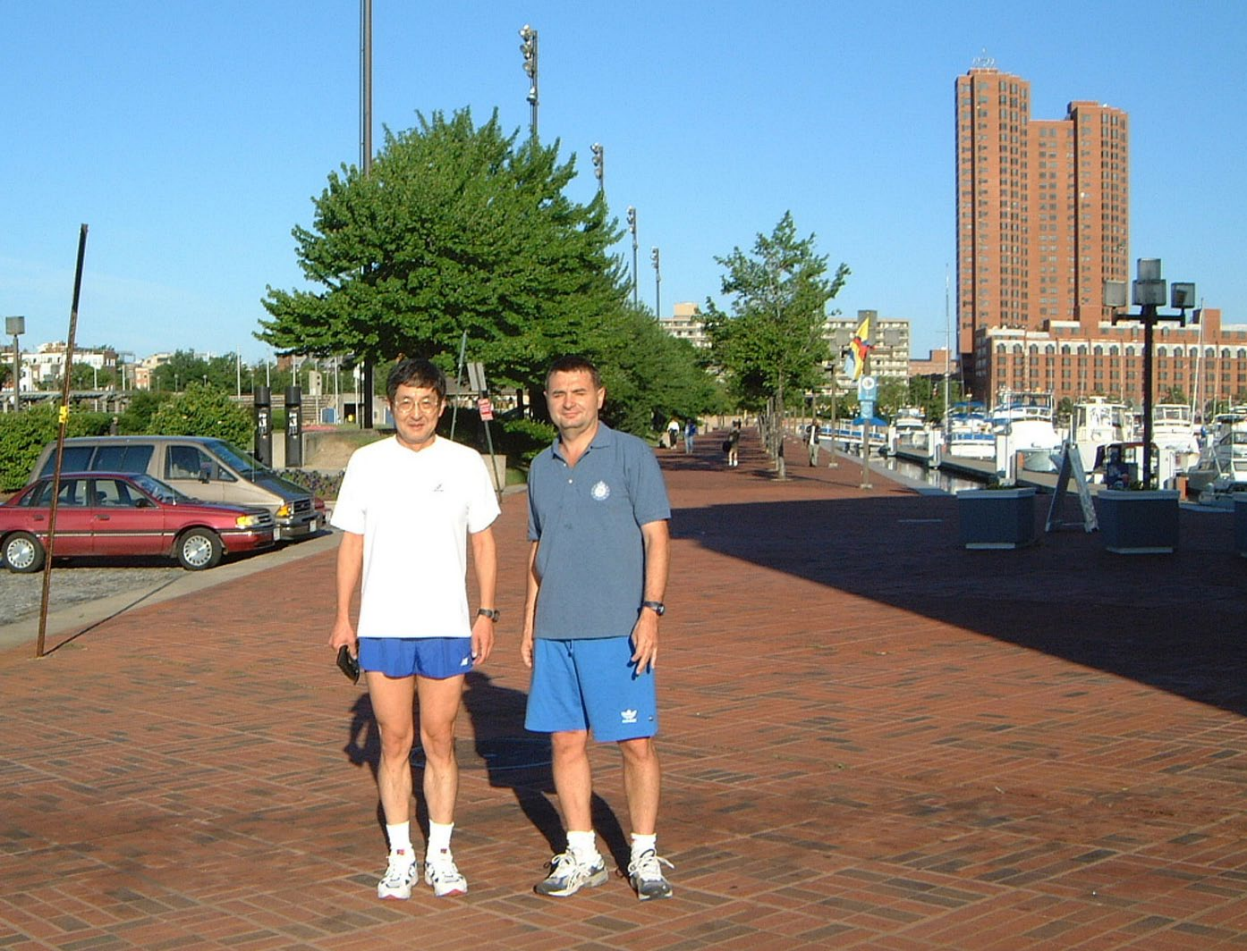


## Zsolt Radak (ZR): Dear Sataro, please tell us something about your childhood and tell us how your father influenced you to go to a biologically-oriented faculty in a university

Sataro Goto (SG): I was born in 1941 about half a year before the beginning of the war between Japan and USA in the Second World War, when the average lifespan of Japanese was less than 50 years, 30 years shorter than now! My family had to move to the countryside from Tokyo, leaving my father who was working in a university. Because the war was so hard to stay in the city and I spent my childhood in the place surrounded by nature and small creatures. I became much interested in those things. After staying there for several years we came back to Tokyo when the war was over. It was quite natural that I entered the faculty related to biology and science later. My father was Professor of Microbiology in the Faculty of Medicine, of The University of Tokyo, and advised me to go on to study in chemically-oriented but health-related faculty rather than medicine, as he recognized importance of chemistry to study biology or medicine in future. I decided to study in the graduate school later belonging to the Laboratory of Microbiology and Biochemistry by the guidance of thoughtful mentor Professor Den'ichi Mizuno who specialized in microbiological physiology. He was interested in multiple disciplines such as origin of life, cancer treatment, biological aging and many others in addition to his expertise of microbiology. I studied, however, in the graduation research for 1 year the structure of proteins by X-ray and infrared ray analysis in the Department of Physical Chemistry of the same faculty. This was a good choice to broaden my background. In the graduate school my mentor, Prof. Den'ichi Mizuno, suggested me to work on the mechanism of aging, quite unexpectedly to my surprise, because no senior graduate student had been involved in the related theme in his laboratory.

At that time, all what I knew about aging was only what was written in a general writing by Ilya Metchnikoff, father of modern gerontology in a sense. My research interest in aging was interrupted for several years, because after getting a PhD degree I went on to study in Karolinska Institute, Sweden, to study cell biology working on mechanism of cell differentiation at the lab of Professor Nils Ringertz in the Institute for Medical Cell Research and Genetics. He was working on the reactivation of the dormant chicken reticulocyte nuclei introduced into the active cells like HeLa cells and mouse fibroblasts so that the dormant nucleus is reactivated in the gene expression. It was a very nice experience because I knew nothing about cell biology. I used cell fusion technique for the research. As an anecdote, before I joined Prof. Ringertz's group, I had applied a post-doctoral position in Dr. John Gurdon, at the University of Oxford who later got the Nobel prize with Shinya Yamanaka. I realized the importance of finding for the regulation of gene regulation in his pioneering work of reprogramming of differentiated cell nuclei of a frog intestinal cells. I got a letter from Gurdon that his laboratory was full and about to move to other institution. I then applied for a position of the laboratory of Ringertz with a fellowship of the International Agency for Research on Cancer. I understood he was working on a similar topic on cell differentiation as Gurdon. Anyway, towards the end of the research in Ringertz's labo, I wished to study molecular biology on the related topics that was the molecular mechanism of cell differentiation with the support of EMBO fellowship under the supervision of Professor Francois Gros with the assistance of Margaret Buckingham in the Department of Molecular Biology at Institute Pasteur in Paris, who was working on the mechanism of muscle cell differentiation. About 2 years of that period were impressive because there were many prominent researchers in the Institute such as Jacques Monod (who had just quitted, however), Francois Jacob who shared the Nobel Prize with Monod for the operon theory of bacterial gene expression, and Jean-Pierre Changeux who was well-known for research on the allosteric proteins and acetylcholine receptor, as you know. I worked on the differentiation of myoblasts obtained from the muscle of bovine fetus. This is one of motivations how I later got into the field related to my current interest of exercise and physical activities. And you see, I met you and then move further into exercise physiology field in aging. It was a very nice experience for me to stay several years in excellent European laboratories.

Some years after returning to Japan I started to work on aging with a small independent team of biochemistry lab at Toho University. Aging research was not popular at that time in Japan. That was one reason why I decided to work on that theme there as my mentor used to tell his students that "do not try to follow popular research".

## ZR: What was your research interest in aging?

SG: I was interested in biochemical and molecular biological mechanisms of aging. Among many possibilities, I decided to work on proteins because of their importance in life but apparently having attracted less interest compared with other potentially important biomacromolecules like membrane lipid and DNA for reason that I did not understand. I first started to work on the Error Catastrophe Theory of Aging as it appeared logical and feasible. As you know, this theory predicted that information transfer from DNA to protein is never perfect possibly causing in the increase in error containing molecules with age. The original idea is said to be proposed by Leslie Orgel but I realized by surveying literatures that the independent similar proposal by Zhores Medvedev is important as well, so I usually cite his report in my writings when referring to the theory. I knew later that he was a very brilliant scientist when he visited my lab in Toho to give a talk on the topic. Anyway, the error theory predicts that the information transfer is not perfect and the errors should naturally occur also in the proteins of information transfer machineries. The errors could propagate by time in life. We decided to work on translation particularly as the process is most directly related to the formation of protein. Let me tell you a bit detail of the process as the theory is not popular now and might attract less interest by young audience but appears historically significant.

As you know, of course, the translation consists of two steps, both of which could give rise to errors in information transfer. The first step is binding of a specific tRNA with the cognate amino acid by catalysis with individual specific aminoacyl tRNA synthetases, and the second step is correct reading of codons (decoding) of mRNA on ribosomes with anticodons of tRNA carrying correct amino acid formed in the first step on ribosomes. We found that in both steps, the fidelity does not change with age up to very old age in mice or rats. So, together with reports by other investigators, we thought that the Error Catastrophe Theory was not valid to explain aging.

In the meantime, literatures were reporting that altered form of proteins increase as animals age. Hungarian scientist Friz Vezar, you may know his name, reported that cross-linking of collagen is increased in rat tail tendons with aging, suggesting that altered forms of protein are possibly a cause of aging. But collagen is an extracellular protein and the finding may not be generalized for other proteins. I was interested in intracellular proteins such as metabolic enzymes which are likely more relevant to physiological functions of a body.

Then, oxidative stress was emerging as a cause of posttranslational alteration of proteins and I was also interested in that. Earl Stadman in NIH of USA was a leader of the field of oxidative modifications of proteins. He and his collaborators developed a chemical method to study the oxidative stress on proteins and found that carbonyls are the most frequently observed type of modifications by oxidation. He established a method to measure carbonyls by the reaction with so called carbonyl reagent 2,4-dinitrophenylhydrazine to measure the products spectrophotometrically. Apart from his chemical method, I was interested in measuring the modified proteins with immunological method. I remembered that when I have learnt in immunology lecture of university course that Karl Landsteiner, Nobel Prize laurate for the discovery of blood type, was able to distinguish different types of isomers in the aromatic ring modified with nitro moieties, because of immunological distinction being very specific. I asked my doctoral student to try to prepare specific antibodies using this classical technique as the Stadman's method of carbonyl measurement is based on the nitration of aromatic rings which should be highly immunogenic. One first oxidizes bovine serum albumin in vitro and then modify them with 2,4-dinitrophenylhydrazine to make it into the hydrazones and then use them as antigens in rabbits. My student got nice specific antibodies.

## ZR: Smart idea. Very good. I know the story, that's the time when I arrived at your lab

SG: Yes, after that, when Rod Levine from Stadman' lab, you know him also, visited us to give a talk and I realized that he had the same idea of producing antibody against the carbonylated proteins to detect them immunochemically. We used to do everything very precisely and took extra time to confirm our findings thus delayed in publishing our results.

At that time, I thought that to have an accumulation of altered forms of protein, it is not just the formation of altered forms, but also due to the reduced degradation of those forms of protein. So, we decided to work on protein turnover in aging. Another reason why I was interested in that was that, again, my mentor Professor Mizuno, had told me that, Rudolf Schönheimer was an unique scientist working in 1930s in Germany and he had published papers and a book,” The Dynamic State of Body Constituents" based on his research in rats using stable isotopes that were discovered in those days and used to label biomolecules in vivo. And this is the first finding of pointing out the importance of turnover of protein and other smaller molecules in animals of which interpretation was incorrectly criticized by Jacques Monod. I will not go into details on this issue here. We studied protein turnover in cells in culture of aging mice. This is not like the study of Hayflick type of in vitro cell senescence but instead we used non-dividing cells isolated from aging mice.

As you know, liver parenchymal cells do not multiply in culture usually. So, we isolated hepatocytes from mice at various ages and cultured them in vitro to study the protein turnover in the cell.

You can use radioactive amino acids to label proteins in culture to study degradation of proteins by pulse labeling and chase experiments. But I did not to use that conventional method. But instead, we introduced proteins into cells in culture by osmotic shock method which was reported then. The reason why that method was better than pulse-chase experiments was that you can modify proteins in vitro as you like, for instance, such as oxidative modifications or any other ways before introduction into cells. You can keep those cells in culture at least for several days with no cell division or obvious harm, for instance, such as decrease in protein synthesis rate that we have tested.

We found that the half-life of protein introduced into the cells from old mice was about 50 percentage longer than in those from younger mice. Age-associated extension of the half-life was also found with the oxidatively modified protein. We therefore concluded that extension of half-life can be one reason of the accumulation of altered proteins with aging. This sort of experiments could not have been done in whole animal study as it was not realistic to have enough number of old animals to study turnover to get statistically significant results.

We further studied the enzyme possibly responsible for the degradation of proteins. It was found that activities of two forms of proteasome, 20S and 26S complexes separated on glycerol gradient centrifugation decreased as animals age.

Around that period, my first foreign post-doc Igor Kurochkin, who came from Ukraine, a part of USSR at that time, made an interesting finding when he was looking for proteolytic enzymes other than proteasome that might be responsible for degradation of oxidized proteins as I thought there might be proteolytic enzymes other than the proteasome. Despite of his original interest in translational fidelity in aging on which we were working, I suggested him to study degradation of oxidized protein on which I had a concern as mentioned above. Let me tell you about Igor's unique study in short.

Using oxidized chicken lysozyme as substrate he found insulin-degrading enzyme (IDE) as a candidate, but it turned out that it was already known to degrade oxidized proteins in rabbit reticulocytes. But his imagination was beyond in that it might degrade amyloid β (Aβ) as well because he speculated that if IDE recognizes β pleated sheet in concentrated insulin why a similar secondary structure in Aβ would not be recognized by the enzyme. We visited a showroom of microscope company to use polarizing microscope to confirm the β-pleated sheet in dense insulin solution. This was how he speculated that IDE might degrade Aβ which was supposed to cause Alzheimer's disease. A very unique idea! Our paper was accepted by FEBS letters after rejection by Nature but not much cited perhaps because we were no body in the community, until Dennis Selkoe, a famous scientist of the study of Alzheimer's disease, replicated our finding. Also, this is probably because most people had been interested in the generation of Aβ in Alzheimer's disease and believed that the increase in Aβ is due to increased generation from the amyloid precursor protein (APP) rather than decrease in degradation. After the Selkoe' s report, many researchers have studied the degradation of Aβ as a mechanism of increase in the notorious peptide in the disease etiology as "A forgotten half" (from the title of International Alz Forum). I was glad of his finding because the degradation of protein had not been focused enough in the history of protein metabolism in general compared to the positive side, the synthesis.

Now I shall move back to the main story of our research. In addition to the mechanisms of aging, I was interested in the intervention of aging that was the caloric restriction or dietary restriction (CR/DR) which is very general way of intervening aging process in different species of animal. Although myself being a graduate of Pharmaceutical Science faculty, I have not thought that supplements or other simple chemicals are good to intervene the complex phenomena of aging to retard the process. Rather, I believed CR/DR can influence basic mechanisms of aging in view of its general impact on aging.

When we started to work on the topic, most of published experiments on CR/DR have been done from early stage of life of animals such that the restriction starts at too young ages, obviously unrealistic if we think of translating results of animal experiments into people if we are to think of growing children being meal restricted by their parents. I therefore decided to use middle-aged animals for the experiments. In our experiments CR/DR was mostly initiated at 20–25 months of age in rats and mice that are equivalent to 40–50 years of age in human. The animals were kept until those ages since such animals were not available commercially. We found that the half-life of the proteins introduced into hepatocytes of the middle-aged animals was reduced to the young animal level and proteasome activity was increased by 2–3 months of CR/DR as examples of possible retardation of aging process.

I did not, however, believe that this is also true in human, because in human, majority of people do not eat as much as they want but rather already restrict themselves in the amount of meal, perhaps due to health education, at least in Japan.

In an on-journal discussion conducted in *Biogerontology,* initiated by Suresh Rattan the Editor-in-Chief on whether CR/DR would also be effective in human, I described that despite potential beneficial results of the regimen, the restricted food intake would not work in human as in animals. Half of the contributors to the discussion among ten participants agreed that the restriction would retard aging and half including myself would not.

In the meantime, you happened to appear in my lab introduced by my friend (Dr. M. Matsuo) working at the Tokyo Metropolitan Institute of Gerontology who gave a talk on aging in a meeting where you were an audience as a postdoctoral fellow. At that time you had just finished graduate course of exercise physiology in a Japanese university and he suggested you to visit me to talk on oxidative stress and aging in which you were interested. Is this story correct?

This was a good timing for me too, because I have had concerns about beneficial mechanisms of habitual exercise as another potential means to intervene in aging. Perhaps, it was 1996 when you joined in our group? There is another story related to this. I was interested in potential mechanism of apparent positive effects of drinking modest amount of liquor (Sake in Japanese: there is a traditional Japanese saying that modest amount of drinking Sake is a best medicine). I thought that a slight chemical damage due to drinking might induce beneficial effects, for instance, acetaldehyde generated as a metabolite of ethanol which can modify proteins and nucleic acids that could upregulate degradation of damaged proteins and repair of modified DNA as a kind of hormesis. I asked my student to test it in cultured cells. This idea was not realized but when you came up to my lab I told you this idea if it can be done in exercise, not in Sake drinking, as exercise might increase oxidative stress and thereby protein damage mimicking the proposed Sake experiment if modest. When you came back to Hungary, you have done experiments on swimming training in rats focusing on oxidative stress. You found that the regimen reduced oxidative damage in brain proteins upregulating proteasome activity and, very interestingly, improved cognitive function in middle-aged animals. That was very important findings that have been cited many times. You know, of course, that we have also shown later that regular treadmill running reduced the oxidative modification in nuclear and mitochondrial DNA in rats which might be a mechanism of beneficial effects of habitual exercise or physical activity.

Since I have collaborated with you, I could have made many connections with researchers in exercise physiology field thanks to you, continuing research after retiring from Toho University, belonging to the Department of Exercise Physiology in Juntendo University (Profs. S. Katamoto, H. Naito) and more recently TMIG (Dr. S. Endo), Japan.

## ZR: Dear Sataro, thank you very much, you made my part very easy. Some short questions at the end of your interview. Can we increase the maximum lifespan? Can we change our mean lifespan?

SG: I don't think the maximum lifespan is extended by any kind of intervention. But you may possibly extend lifespan up to close to the genetically fixed maximum level. We should not, however, expect extending the maximum unless you can change your genes by, for instance, introducing genes of extremely long-lived animals such as blue whale, Galapagos giant tortoise and naked mole rat, if anything can be hoped, without altering functions of most genes which we already have. But this would not be realistic even far future.

## ZR: But how about Yamanaka factors?

SG: The contribution of Yamanaka in biology and medicine is obviously great but you should not expect too much in aging such as "treatment" of biological aging in general as some scientists claim that aging is curable by a sort of stem cell therapy, I think.

Some cell therapies may influence the certain functions but perhaps not in many tissues. It would not significantly extend health-span of the organisms, because obviously you would not be able to rejuvenate all cells in your old body by any kind of stem cell therapy. It should be more realistic to try to reduce the rate of functional decline with advancing age by physically active life and adequate daily food intake.

Before finishing this interview let me add a few words what we can do as a researcher in aging societies. After retiring from active research some 15 years ago, I now and then have given talks and written web articles for citizens on scientific knowledge of aging because a lot of incorrect or biased information on aging is spreading through mass-media networks, popular journals and books, particularly in aged society like Japan. I believe that this sort of education of citizens is an important task of scientists to assist people judge such complex information by themselves.

Another issue related to this is that we scientists should try to tell citizens not believe in what prominent scientists claim on what he or she is supposed to know well, or sometimes does not know well on specific issues despite he or she would be an expert of a field.

I cite an example on the telomere. The telomere is believed to be a key player of aging but the reason for the Nobel Prize Elizabeth Blackburn got has nothing to do with aging as I have described this in my blog in Japanese. Nevertheless, a famous book coauthored by her apparently claim that it has. Moreover, the book was strongly recommended by Erick Kandel who had gotten the Nobel Prize on neural transmission as a book of most important contribution on the mechanism of aging, giving a wrong impression for amateur audience as such. I realize, however, of course, that Blackburn's discovery on the telomere function is very great.

On this occasion, let me add a bit about respected Denham Harman, the original advocate of the Free-radical Theory of Aging. I knew him through Ken Kitani who was my good friend. I wrote an obituary for admiring Denham in Biomedical Gerontology (in Japanese), official journal of the Japan Society, describing not just his scientific contributions but also his personality of which I have known through acquaintance with him.

You know, he is one of the most well-known scientists in aging research. But the number of his publication was relatively few, writing papers mostly with his single name stressing his own idea, apparently not being interested in publishing many papers with worldwide potential collaborators that he could have. This is not because he was stubborn as, you can see, he has originated the International Association of Biomedical Association (IABG) and continued to support it for the development of aging research in general. I have participated since the modest first meeting in New York with Ken in 1985. Denham even organized the 9th meeting by himself with his wife Helen at the age of 85, proving his belief and vitality in aging research. He described an episode with a Russian lady who was an immigrant to USA and continued to work until 104 years of age to whom Denham himself had tried to follow as she believed in working until late in life is important for healthy longevity. Actually, Denham had worked in his office of Nebraska University as professor emeritus until very close of his death at 97 in 2014, with regular jogging even though he had perhaps believed in beneficial effect of antioxidants for healthy aging as the advocate of the Theory. (The details of the story can be referred to in the interview article of Denham by Ken Kitani, Biogerontology 4: 401–412, 2003).

ZR: Dear Sataro, thank you so much, and let’s repeat this interview 20 years later!

